# Emerging Role of the NLRP3 Inflammasome in the Onset of Oral Diseases and Its Potential as a Therapeutic Target

**DOI:** 10.3390/ijms27021098

**Published:** 2026-01-22

**Authors:** Mohammad Ibtehaz Alam, Fatima Farhana, Eiko Sakai

**Affiliations:** 1 Department of Periodontology & Oral Pathology, University Dental College, Dhaka 1217, Bangladesh; mohammad_du49@yahoo.com; 2 Department of Endodontics, Center for Regenerative Medicine and Skeletal Development, University of Connecticut Health, Farmington, CT 06030, USA; 3 Department of Prosthodontics, University Dental College, Dhaka 1217, Bangladesh; fatimafarhana.2011@gmail.com; 4 Department of Medical Research and Development for Oral Disease, Nagasaki University Graduate School of Biomedical Sciences, Nagasaki 852-8588, Japan

**Keywords:** innate immune system, NLRP3 inflammasome, interleukin-1β, interleukin-18, GSDMD, oral diseases, periodontitis, pulpitis, PAMPs, DAMPs

## Abstract

Growing evidence suggests that persistent oral infectious diseases (OIDs) contribute to systemic disease, highlighting the importance of understanding their pathogenic mechanisms. Conventional dental treatments, primarily mechanical debridement, surgical intervention, or antimicrobial therapy, often struggle to fully control inflammation or prevent progressive tissue destruction. The nucleotide-binding oligomerization domain, leucine-rich repeat, and pyrin domain-containing 3 (NLRP3) inflammasome is a key regulator of innate immunity, mediating the maturation of proinflammatory cytokines (IL-1β and IL-18) and the pyroptosis-inducing protein gasdermin D. Dysregulated or excessive activation of NLRP3 contributes to the initiation and progression of major oral diseases, including periodontitis, peri-implantitis, pulpitis, and oral mucosal inflammation. Despite growing interest in NLRP3, comprehensive and up-to-date reviews integrating its pathogenic mechanisms and therapeutic potential remain limited. This review summarizes current and past evidence on the role of the NLRP3 inflammasome in oral disease development, highlights emerging pharmacological strategies, and outlines future research directions. Existing studies demonstrate that microbial components and danger signals from injured tissues activate NLRP3, thereby amplifying inflammation, tissue degradation, and bone resorption. Preclinical studies indicate that inflammasome inhibitors and several natural compounds reduce tissue damage; however, their clinical translation remains limited. These findings emphasize the need for deeper understanding of NLRP3-mediated pathways, with translational and clinical research offering promising therapeutic opportunities for oral diseases.

## 1. Introduction

Oral inflammatory diseases (OID), including periodontitis, peri-implantitis, pulpitis, periapical lesions, oral mucosal lesions, and other chronic oral disorders, have become a major global health concern due to their treatment limitations and associated economic burden [1,2]. These diseases were once considered to affect only oral health and quality of life; however, growing evidence indicates that persistent OID, such as periodontitis, also contributes to the development of systemic conditions, including diabetes, osteoporosis, rheumatoid arthritis, Alzheimer’s disease, and cardiac infections. This underscores the importance of understanding the mechanisms underlying OID pathogenesis [3,4,5]. To manage oral and dental diseases, standard dental treatments typically focus on mechanical debridement, surgical excision, or antimicrobial therapies. Unfortunately, these conventional approaches often fail to adequately control inflammation or prevent the gradual destruction of oral tissues [6]. Such findings highlight the need for a deeper understanding of the molecular and immunological mechanisms regulating oral inflammation.

The innate immune system plays a pivotal role in maintaining host physiological balance. The nucleotide-binding oligomerization domain–leucine-rich repeat (LRR)– and pyrin domain–containing protein 3 (NLRP3) inflammasome, a multiprotein complex within the innate immune system, is a key component in host defense [7]. Although NLRP3 plays a crucial role in protecting the body from illness, excessive or uncontrolled activation of this protein can lead to tissue injury and adverse health outcomes [8]. Upon sensing microbiota-derived pathogen-associated molecular patterns (PAMPs) or danger-associated molecular patterns (DAMPs) released from damaged tissues, the NLRP3 inflammasome assembles and recruits pro–caspase-1, which is then cleaved into active caspase-1. This process mediates the maturation of proinflammatory cytokines IL-1β and IL-18, as well as the activation of gasdermin D (GSDMD), a pore-forming protein responsible for inducing pyroptotic cell death [7,9].

Growing scientific evidence confirms the critical involvement of the NLRP3 inflammasome in various oral diseases, including periodontitis, pulpitis, periapical lesions, inflammatory disorders of the oral mucosa, and even the development and progression of oral squamous cell carcinoma (OSCC) [10,11,12,13,14]. This indicates that NLRP3 may serve as a potential therapeutic target and biomarker for these conditions [9]. Although research on the role of NLRP3 in oral diseases is expanding, the available data remain fragmented, and comprehensive reviews on this topic are scarce. This highlights the need for an updated synthesis integrating the role of NLRP3 in OID, its underlying mechanisms of action, and its potential as a therapeutic target. Therefore, this review integrates current understanding of NLRP3 inflammasome activation in oral diseases, its impact on disease progression, and emerging therapeutic prospects.

The objective of this review is to address this gap by consolidating preclinical and clinical evidence, elucidating molecular mechanisms, and outlining future research directions regarding the interplay between the NLRP3 inflammasome and oral inflammatory diseases to enhance oral and dental health.

## 2. Structure and Activation of the NLRP3 Inflammasome

### 2.1. Structure of the NLRP3 Inflammasome

**Figure 1:**
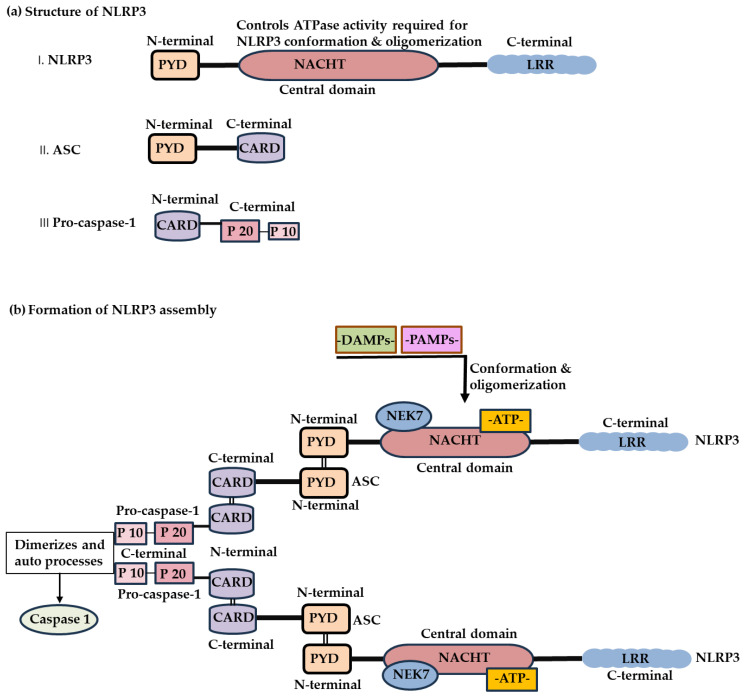
Schematic illustration of the structural organization of the NLRP3 inflammasome. (**a**) Domain architecture of the NLRP3 inflammasome components. NLRP3 includes an N-terminal PYD, a central NACHT domain responsible for ATP-dependent oligomerization, and C-terminal LRRs. ASC contains an N-terminal PYD and a C-terminal CARD, while pro–caspase-1 comprises an N-terminal CARD and C-terminal catalytic p20 and p10 subunits. (**b**) Architecture of the active NLRP3 inflammasome assembly. Upon sensing pathogen- or danger-associated molecular patterns, NLRP3 undergoes conformational changes, allowing NEK7 binding and NACHT-mediated oligomerization. This process enable NLRP3-ASC interacting via their PYDs to recruit ASC. Subsequently, ASC recruits pro-caspase-1 through interactions between CARDs. The pro-caspase-1 domain (p20/p10) then dimerizes and undergo auto-processes to generate active caspase-1.

The NLRP3 inflammasome consists of three primary components: the sensor protein NLRP3, the adaptor molecule apoptosis-associated speck-like protein containing a caspase-1 recruitment domain (ASC), and the effector enzyme pro–caspase-1 [7,15]. The NLRP3 protein comprises C-terminal LRRs that participate in ligand sensing and autoinhibition, a central nucleotide-binding and oligomerization (NACHT) domain that facilitates adenosine triphosphate (ATP)–dependent oligomerization, and an N-terminal pyrin domain (PYD) that mediates its interaction with ASC [16]. In contrast, ASC contains an N-terminal PYD and a C-terminal caspase activation and recruitment domain (CARD), whereas pro–caspase-1 consists of an N-terminal CARD and C-terminal catalytic subunits (p20 and p10) [15,16] (Figure 1).

### 2.2. Activation of the NLRP3 Inflammasome

**Figure 2:**
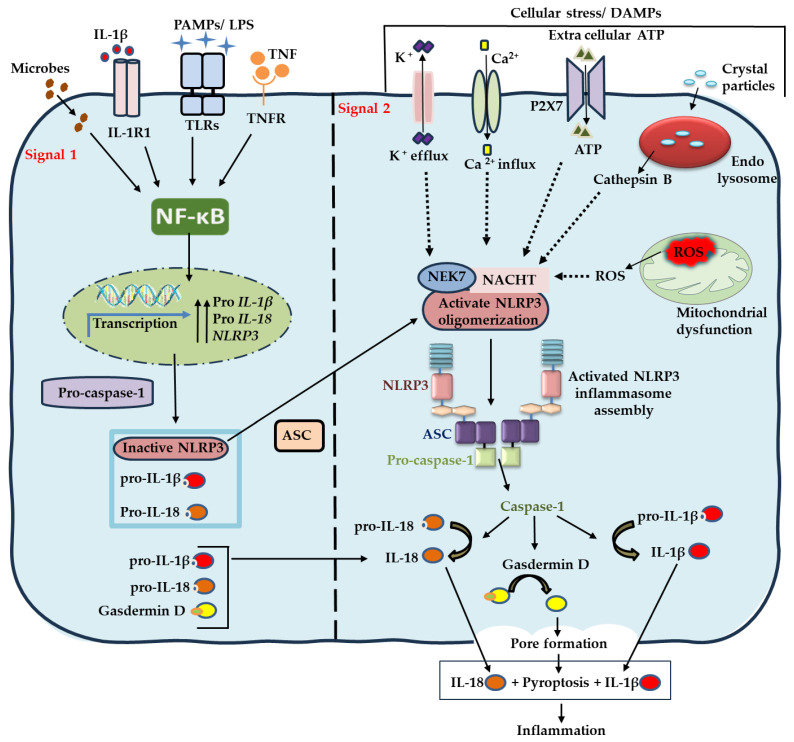
Two-signal activation model of the NLRP3 inflammasome. NLRP3 activation requires two sequential signals. Signal 1 (priming) is mediated by IL-1R1, TLRs, or TNFR upon ligand recognition, which activates NF-κB and induces transcription of NLRP3, pro–IL-1β, and pro–IL-18. Signal 2 (activation) is triggered by various PAMPs from microbiome and DAMPs released from damaged tissue such as potassium efflux, calcium influx, ATP–P2X7 receptor signaling, lysosomal rupture, and mitochondrial ROS—which activate the conformation and oligomerization of NLRP3. Activated NLRP3 then recruits ASC and pro–caspase-1 to form the inflammasome complex, leading to caspase-1 activation. Active caspase-1 cleaves pro–IL-1β and pro–IL-18 into their mature forms and processes GSDMD, which forms pores in the plasma membrane to facilitate the secretion of IL-1β and IL-18.

Activation of the NLRP3 inflammasome occurs through a canonical two-step process: priming and activation (Figure 2) [17,18,19].

Priming (Signal 1): This step increases the amount and readiness of the inflammasome components required for future NLRP3 assembly formation. Bacterial components such as lipopolysaccharide (LPS), proinflammatory cytokines including tumor necrosis factor alpha (TNF-α), or other agonists engage surface or endosomal pattern recognition receptors—such as Toll-like receptors (TLRs), nucleotide-binding and oligomerization domains (NODs), and cytokine receptors—to initiate this process. These receptor–ligand interactions activate the nuclear factor kappa B (NF-κB) signaling pathway, resulting in the transcriptional upregulation of NLRP3, pro–IL-1β, and pro–IL-18 [15,17]. The priming step also facilitates deubiquitination and other molecular modifications that render NLRP3 assembly competent, whereas post-translational modifications such as phosphorylation, sumoylation, and ubiquitination maintain NLRP3 in an inactive state [18].

Activation (Signal 2): Upon sensing PAMPs (i.e., Leukotoxin A, LPS), DAMPs, or cellular stressors—such as extracellular ATP, potassium efflux, calcium influx, mitochondrial ROS generation, and lysosomal rupture—NLRP3 undergoes a conformational rearrangement through its interaction with NIMA-related kinase 7 (NEK7). NEK7 is an essential regulatory protein that facilitates NLRP3 oligomerization and promotes inflammasome assembly. This conformational change initiates ATP-dependent oligomerization of the central NACHT domain, which enables PYD-PYD interactions between NLRP3 and the adaptor protein ASC. These interactions allow ASC recruitment and subsequent CARD–CARD interactions between ASC and pro–caspase-1, leading to the formation of an active inflammasome complex and the autocatalytic conversion of pro–caspase-1 into its active form, caspase-1. Active caspase-1 then cleaves pro–IL-1β and pro–IL-18 into their active forms [7,15]. In addition, caspase-1 cleaves GSDMD, whose N-terminal fragment inserts into the plasma membrane to form pores that mediate pyroptotic cell death [17,18,19].

In oral diseases, multiple cellular stressors can act as DAMPs that promote NLRP3 activation. Moreover, virulence factors (LPS, gingipains, LtxA) from microbial pathogens such as *Fusobacterium nucleatum*, *Porphyromonas gingivalis* and *Aggregatibacter actinomycetemcomitans* can sustain chronic NLRP3 inflammasome activation by providing both priming and activation signals, thereby perpetuating inflammation and oral tissue destruction [19,20,21].

## 3. Role of the NLRP3 Inflammasome in Oral and Dental Diseases

**Figure 3:**
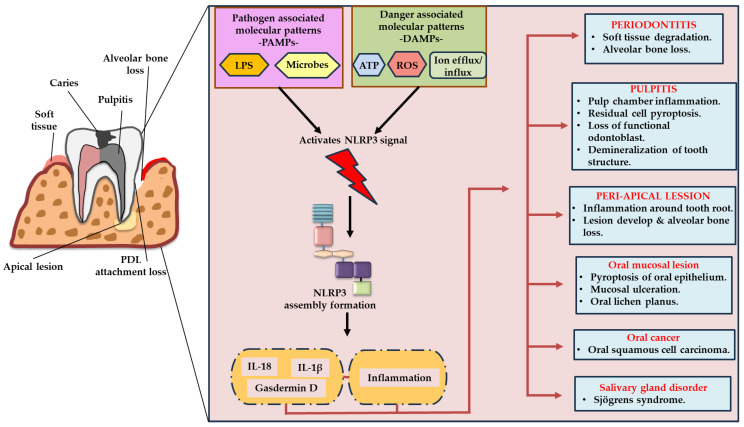
Schematic illustration of the role of the NLRP3 inflammasome in mediating major oral inflammatory diseases. PAMPs derived from the oral microbiota and DAMPs released from injured tissues activate the NLRP3 inflammasome in epithelial, connective tissue, immune, and other resident cells of the oral cavity. This activation induces pyroptosis and the release of proinflammatory cytokines, which further amplify inflammation and play a pivotal role in the onset and progression of major oral inflammatory diseases such as periodontitis, pulpitis, periapical lesions, orthodontic and prosthodontic treatment complications, oral mucosal disorders, salivary gland inflammation, and OSCC. PAMPs: pathogen-associated molecular patterns; DAMPs: danger-associated molecular patterns.

NLRP3 acts as a frontline innate immune sensor in oral tissues, including the oral epithelium, gingival fibroblasts, dental pulp cells, odontoblasts, and immune cells. While controlled activation provides host protection, persistent or excessive NLRP3 activity contributes to chronic inflammation, and, both soft and hard tissue destruction, leading to the onset and progression of OID such as periodontitis, pulpitis, periapical lesions, oral mucosal disorders, and OSCC [10,11,12,13] (Figure 3).

### 3.1. Periodontitis

Periodontitis is a chronic inflammatory oral disease characterized by the progressive destruction of tooth-supporting tissues. Inflammation in periodontal tissues typically originates from oral biofilm deposits, such as plaque and dental calculus (DC), which provoke an excessive host immune response to subgingival microbiome dysbiosis [22,23]. In recent years, the NLRP3 inflammasome has been identified as a crucial molecular link between microbial stimuli and destructive periodontal inflammation leading to alveolar bone loss [10,20]. PAMPs, such as bacterial LPS, gingipains, LtxA derived from periodontopathic bacteria contained in dental deposits, initiate TLR/NF-κB–dependent priming of NLRP3. This priming is followed by inflammasome activation triggered by DAMPs, including DC particles, extracellular ATP, ROS, potassium efflux, mitochondrial dysfunction, and lysosomal rupture in gingival epithelial cells, fibroblasts, and infiltrating macrophages [10,23,24]. This signaling cascade results in caspase-1 activation, which in turn mediates the cleavage of pro–IL-1β and pro–IL-18 into their mature forms [9]. These cytokines amplify periodontal inflammation by promoting neutrophil hyperresponsiveness and excessive neutrophil extracellular trap (NET) formation [25], driving macrophage polarization toward the M1 phenotype [26], and disrupting Th17/Treg balance. They also stimulate matrix metalloproteinase (MMP) activity and enhance prostaglandin synthesis [27,28,29,30], collectively contributing to connective tissue degradation, loss of periodontal ligament (PDL) attachment, and accelerated osteoclast differentiation [10,31]. Beyond cytokine maturation, NLRP3 activation induces GSDMD cleavage and pyroptotic cell death in macrophages, epithelial cells, and PDL cells. This results in cell swelling, lytic cell death, and micro-ulceration formation [32,33]. The formation of GSDMD pores facilitates the release of DAMPs such as ATP, high-mobility group box 1 (HMGB1), and mitochondrial DNA (mtDNA), which further enhance NLRP3 activation, establishing a self-amplifying inflammatory loop. This loop accelerates junctional epithelium breakdown, allows deeper microbial invasion, and exacerbates tissue destruction, expanding the role of NLRP3 beyond inflammation to direct cell loss [32,34,35]. The transition toward the pocket epithelium and apical area is further intensified by IL-1β–driven epithelial hyperplasia, decreased antimicrobial peptide expression, and persistent infiltration of neutrophils and macrophages, all of which perpetuate periodontal tissue destruction [30]. This cycle of inflammasome activation and pyroptotic cell death is now recognized as a central pathogenic mechanism in periodontal breakdown. Clinical studies have consistently demonstrated elevated levels of NLRP3, caspase-1, IL-1β, and IL-18 in the gingival tissues and gingival crevicular fluid of patients with periodontitis, correlating with attachment loss, probing depth, and bleeding on probing [36,37].

There is a strong correlation between NLRP3-dependent inflammatory signaling and periodontitis- bone loss. Mature IL-1β, generated through NLRP3 inflammasome activation, promotes the expression of receptor activator of nuclear factor kappa B ligand (RANKL) in stromal, PDL, and osteoblast-lineage cells [38]. In the presence of RANKL, mature IL-1β acts synergistically to accelerate osteoclastogenesis by upregulating nuclear factor of activated T cells cytoplasmic 1 (NFATc1) and cathepsin K (CtpK) expression [39,40]. Correspondingly, compared with wild-type controls, NLRP3- or caspase-1–deficient mice exhibit significantly less alveolar bone loss in ligature-induced periodontitis models, underscoring the crucial role of inflammasome signaling in periodontal bone resorption [40,41]. Furthermore, recent findings revealed that DC particles function as DAMPs, inducing IL-1β production through an NLRP3-dependent pathway in macrophages and exacerbating osteoclastogenesis [42]. These results confirm that NLRP3 acts as an active mediator of alveolar bone resorption rather than a passive participant. Interestingly, in vitro evidence suggests that NLRP3 may exert context-dependent, dual roles in bone biology. While NLRP3 promotes infection-induced osteoclastogenesis via IL-1β signaling, under sterile conditions it may restrict excessive osteoclast differentiation by inducing pyroptosis in osteoclast precursors—indicating a potential homeostatic function in physiological bone remodeling [43].

Collectively, these findings highlight the crucial and multifaceted role of the NLRP3 inflammasome in maintaining periodontal health and contributing to disease. This dual nature positions NLRP3 as a promising therapeutic target and emphasizes the importance of developing context-specific strategies to modulate inflammasome activity in periodontal homeostasis.

### 3.2. Pulpitis and Periapical Lesions

The NLRP3 inflammasome plays a pivotal role in coordinating the inflammatory response in dental pulpitis and promoting periapical lesion progression [44,45]. Following dental caries, tooth cracks or trauma, bacteria infiltrate the dentin and subsequently reach the pulp chamber via dentinal tubules, exposing the pulp to the oral environment. Microbial invasion of the pulp chamber activates TLR2/4–NF-κB signaling, which mediates the priming step of NLRP3 activation in odontoblasts, pulp fibroblasts, endothelial cells, dendritic cells, and resident macrophages [44,46]. Odontoblasts located at the dentin–pulp interface serve as the first line of defense, recognizing invading bacteria and upregulating NLRP3 and pro–IL-1β mRNA, thereby acting as the primary site for inflammasome priming within the pulp [47]. Beyond microbial stimuli, hypoxia and metabolic stress generated during pulpal injury, along with DAMPs released from damaged or dying odontoblasts, further promote NLRP3 assembly within the confined pulp chamber. This highlights the involvement of both pathogen- and danger-associated signals in inflammasome activation within pulp tissue [11,44,48]. Once activated, NLRP3 induces a markedly proinflammatory microenvironment through caspase-1–dependent maturation of IL-1β and IL-18, resulting in increased vascular permeability, leukocyte recruitment, and progressive pulp tissue degradation [44,48,49]. Moreover, NLRP3 activation enhances the production of chemokines such as C-C motif chemokine ligand 2 (CCL2), C-X-C motif chemokine 8 (CXCL8), and CCL20, as well as MMPs, which accelerate neutrophil and monocyte infiltration. This amplifies tissue breakdown and contributes to necrotic progression. The accumulation of necrotic debris supports bacterial proliferation and perpetuates inflammasome activation, leading to a chronically infected necrotic pulp if untreated [25,50,51]. In addition, NLRP3 activation induces the release of secondary mediators such as prostaglandin E2 (PGE2) and TNF-α, which exacerbate inflammation and sensitize nociceptors including transient receptor potential vanilloid 1, transient receptor potential ankyrin 1, and acid-sensing ion channel 3, thereby promoting hyperalgesia in pulpitis [50,52].

Persistent pulp infection and microbial invasion can extend lesions into the periapical tissues, where NLRP3 activation drives periapical bone resorption via the IL-1β–RANKL–osteoclast axis [53,54]. IL-1β enhances osteoclast differentiation and promotes periapical bone resorption by upregulating RANKL expression in T lymphocytes and stromal cells [26,54,55]. Inflamed pulp and periapical tissues exhibit elevated expression of NLRP3, caspase-1, IL-1β, and IL-18, with their levels strongly correlating with lesion size and clinical severity [48,56]. The pathogenic role of NLRP3 is further supported by experimental inhibition studies showing that blocking NLRP3 or caspase-1 significantly reduces pulpal inflammation and attenuates periapical bone destruction in animal models [45,48]. Moreover, persistent NLRP3 activity within the pulp chamber causes continuous damage to odontoblasts via GSDMD which may lead to reduced tertiary dentin formation and impaired regenerative capacity of odontoblasts.

Collectively, these findings demonstrate that the NLRP3 inflammasome plays a central role in mediating pulpitis and accelerates the transition from reversible pulpal injury to chronic pulpitis and periapical inflammation. Therefore, targeting NLRP3 signaling represents a promising therapeutic strategy to improve endodontic treatment outcomes.

### 3.3. NLRP3 Inflammasome in Orthodontic and Prosthodontic Treatment Complications

Orthodontic tooth movement (OTM) and prosthodontic procedures induce mechanical, chemical, and biological disturbances within periodontal tissues [57,58]. These perturbations activate innate immune sensing pathways in resident periodontal cells and infiltrating leukocytes. Among these pathways, the NLRP3 inflammasome has emerged as a critical mediator linking mechanical stress or stimuli derived from orthodontic/prosthodontic treatment and bacterial endotoxins to mediate the local inflammation and tissue injury [59,60]. Such responses often lead to common post-treatment complications including pain, root resorption, delayed healing, peri-implant mucositis, peri-implantitis, and inflammation.

NLRP3 activation serves as a key mechanistic connection between orthodontic force and local sterile inflammation, contributing to both pain induction and bone remodeling. Orthodontic mechanical loading generates compression- and tension-dependent stress within the PDL and alveolar bone, which promotes local hypoxia, induces extracellular ATP release, and increases mitochondrial ROS accumulation in macrophages, PDL cells, and other resident periodontal cells. These stimuli, together with local TLR signaling, collectively trigger inflammasome assembly and promote caspase-1–dependent maturation of IL-1β and IL-18. Moreover, cyclic or compressive mechanical stress can directly activate NLRP3 in these cells [57,59]. A controlled, transient NLRP3 response facilitates physiological osteoclast recruitment during tooth movement. However, excessive or prolonged mechanical force—or the coexistence of bacterial infection—intensifies NLRP3 signaling and upregulates RANKL expression in PDL fibroblasts and osteoblasts, thereby enhancing osteoclast differentiation. This contributes to orthodontic pain, periodontal inflammation, and external apical root resorption [61,62]. Experimental studies have demonstrated that inflammatory tissue damage is markedly reduced when NLRP3 or IL-1β signaling is inhibited in NLRP3^−/−^ mice; interestingly, OTM progression is delayed in NLRP3^−/−^ mice compared with wild-type (WT) controls [63]. These findings suggest that applying controlled orthodontic force combined with proper oral hygiene maintenance is crucial for ensuring biologically safe, NLRP3-mediated orthodontic treatment.

Moreover, prosthodontic complications arise through similar mechanisms, in which abundance of *Porphyromonas gingivalis*, *Fusobacterium vincentii*, *Porphyromonas endodontalis*, *Prevotella oris*, *Treponema denticola*, and *Tannerella forsythia* in peri-implant biofilms significantly associated with initiation and progression of pathogenesis [2]. Presence of these bacteria can provide both priming and activation signal, while titanium-corroded particles from implant surfaces cause lysosomal destabilization, leading to robust NLRP3 activation in peri-implant macrophages. This activation upregulates IL-1β production and accelerates marginal bone resorption. Additionally, cobalt, nickel, chromium, and titanium ions released from dental alloys or implant materials induce ROS generation and mitochondrial stress [2,60,64]. Although extensive research has explored peri-implantitis and its association with NLRP3 inflammasome activation, evidence remains limited regarding how other dental prostheses—such as over contoured crowns and rough denture surfaces—contribute to NLRP3-mediated inflammatory responses. Some studies suggest that mechanical trauma from ill-fitting dentures and microbial metabolites, particularly *Candida*-associated DAMPs, further activate NLRP3 in oral mucosal epithelial and immune cells, thereby promoting soft tissue inflammation and denture stomatitis [65,66].

Collectively, these findings indicate that the NLRP3 inflammasome serves as a key molecular link integrating microbial and mechanical cues that drive both hard and soft tissue complications in suboptimal orthodontic and prosthodontic treatment contexts.

### 3.4. Oral Mucosal Diseases

The NLRP3 inflammasome functions as a double-edged sword within the oral mucosa, detecting chemical, microbial, and endogenous danger signals in the epithelial and lamina propria layers. Under acute conditions, NLRP3 activation contributes to protective host defense by inducing pyroptosis to eliminate infected or damaged cells, thereby maintaining mucosal integrity and facilitating pathogen clearance [67]. Experimental models have shown that impaired NLRP3 activation in oral epithelial cells compromises immunity against opportunistic pathogens such as *Candida* species [68].

Conversely, chronic, dysregulated, or recurrent activation of NLRP3 can have detrimental effects. In keratinocytes and infiltrating immune cells—particularly T lymphocytes—persistent microbial components from the oral microbiota or continuous cellular stress amplify NLRP3 inflammasome activation, promoting sustained secretion of IL-1β and IL-18. This sustained cytokine release drives chronic mucosal inflammation, immune cell infiltration, and epithelial injury. These events contribute to mucosal erosion, ulceration, and epithelial barrier dysfunction, as observed in erosive and atrophic forms of oral lichen planus (OLP) [69,70]. Recent in vitro and ex vivo studies have demonstrated a strong positive correlation between Galectin-3 expression and NLRP3, ASC, caspase-1, and IL-1β levels in OLP lesions, suggesting that Galectin-3 may serve as an upstream regulator of NLRP3 inflammasome activation during OLP progression [71].

However, the role of NLRP3 in other oral mucosal disorders, including recurrent aphthous stomatitis (RAS) and oral leukoplakia (OLK), remains unclear. A previous case study investigated the association between NLRP3 gene polymorphisms and RAS in a Czech population; however, the absence of functional analyses and the sample size limit the validity of this association [72]. Further research integrating genetic studies with functional inflammasome assays in larger cohorts is warranted to validate NLRP3’s involvement in the pathogenesis of RAS. Moreover, few studies have evaluated NLRP3 activation in human oral mucosal tissues using direct measures such as caspase-1 cleavage, IL-1β and IL-18 quantification, or pyroptosis markers. Therefore, although NLRP3 represents a biologically plausible mediator of mucosal inflammation and epithelial dysplasia, its specific role in RAS, OLK, and other oral mucosal diseases remains hypothetical, underscoring the need for comprehensive human-based investigations involving tissue biopsies, inflammasome profiling, and longitudinal clinical follow-up.

### 3.5. Oral Cancer and Salivary Gland Disorders

OSCC is the most prevalent epithelial malignancy of the oral cavity and is primarily driven by chronic inflammation. In the oral mucosa, persistent irritation from tobacco, alcohol, and betel quid use; chronic infections (e.g., *Candida* species); mechanical trauma; or long-standing premalignant lesions establish a chronic inflammatory milieu in which innate immune sensors—including the NLRP3 inflammasome—undergo repeated activation [13,73]. This sustained engagement connects persistent innate immune signaling to DNA damage, immune dysregulation, and the development of a tumor-permissive microenvironment that facilitates tumorigenesis. Microbial PAMPs, chemical irritants from tobacco, cytokines secreted by infiltrating immune cells such as TNFα *and* IFNs, as well as cell-intrinsic stressors (including oncogene activation and metabolic stress), collectively enhance the expression of NLRP3, pro-IL-1β and pro-IL-18 in keratinocytes, stromal cells, and myeloid cells. This establishes a transcriptionally primed state predisposed to inflammasome activation. Subsequently, cellular stress and injury—such as mitochondrial dysfunction, ROS accumulation, K^+^ efflux, extracellular ATP release from injured cells, lysosomal rupture, and metabolic alterations (e.g., increased lactate and reduced pH)—along with DAMPs from necrotic tumor areas, promote NLRP3 oligomerization, assembly of the NLRP3–ASC–caspase-1 complex, and secretion of IL-1β and IL-18 [13,74,75]. These NLRP3-mediated cytokines foster a pro-tumorigenic microenvironment. IL-1β upregulates vascular endothelial growth factor expression and induces endothelial activation, thereby promoting neovascularization and supporting tumor expansion [76,77]. Additionally, IL-1β recruits neutrophils and monocytes and stimulates the differentiation of tumor-associated macrophages (TAMs) with immunosuppressive and proangiogenic phenotypes [78]. Moreover, IL-1β–stimulated MMPs degrade the extracellular matrix, thereby facilitating cancer cell invasion. Collectively, these processes accelerate the transition from dysplasia to invasive OSCC [76,77,79]. In certain contexts, tumor cells themselves express functional inflammasome components. NLRP3 activation within tumor cells enhances proliferative and survival signaling through IL-1R–mediated autocrine and paracrine loops, promotes epithelial–mesenchymal transition (EMT), and increases resistance to apoptosis [80]. Conversely, pyroptotic death of tumor cells releases tumor-associated antigens and DAMPs, which stimulate antitumor immune responses [81]. However, persistent IL-1β signaling augments tumor invasiveness and intravasation and contributes to the formation of pre-metastatic niches by mobilizing bone marrow–derived cells and promoting extracellular matrix remodeling [82]. These effects collectively increase the metastatic potential of tumors characterized by chronic inflammasome activation in OSCC [83]. Experimental inhibition of NLRP3 or caspase-1 has been shown to suppress OSCC cell migration, invasion, and proliferation, underscoring the contribution of NLRP3 inflammasome signaling to tumor progression [77,84,85]. Comparisons between OSCC tissues and normal oral mucosa consistently reveal elevated expression of NLRP3, caspase-1, and IL-1β, which correlate with advanced tumor stage, lymph node metastasis, and poor clinical prognosis [74,85,86]. Nevertheless, some studies suggest that NLRP3 may exert antitumor effects by promoting CD8^+^ T-cell infiltration or inducing pyroptotic killing of cancer cells [87].

NLRP3-driven inflammation contributes to glandular epithelial injury and secretory dysfunction in salivary gland disorders such as Sjögren’s syndrome (SS) [88]. In SS, autoantigen-containing immune complexes, K^+^ efflux, upregulated purinergic receptor P2X7 (P2X7R) expression, and accumulation of ROS activate NLRP3 in salivary gland epithelial cells and infiltrating macrophages. This activation promotes IL-1β–mediated acinar cell apoptosis, salivary hypofunction, and lymphocytic infiltration [88,89]. The focus score and severity of xerostomia strongly correlate with elevated NLRP3 and caspase-1 expression levels in the labial salivary glands of patients with SS. Furthermore, NLRP3 activation is triggered by mitochondrial dysfunction and DNA damage–associated DAMPs, which amplify IL-1β–driven acinar cell death and fibrosis, whereas pharmacological inhibition of NLRP3 partially restores salivary flow in animal models [89,90,91]. Interestingly, DAMP-induced NLRP3 activation in obstructive and chronic sialadenitis has been reported to induce macrophage recruitment, polarization, and glandular tissue remodeling [92,93].

Overall, these findings demonstrate that NLRP3-mediated inflammation exacerbates both oral epithelial tumorigenesis and inflammatory destruction of salivary glands. However, limited evidence also suggests that NLRP3 may exert context-dependent protective effects under specific conditions.

## 4. Therapeutic Potential of Targeting the NLRP3 Inflammasome

The NLRP3 inflammasome has been identified as a central regulator in the initiation and progression of OID, generating substantial interest in the development of NLRP3-targeted therapeutic strategies. Consequently, researchers have investigated diverse pharmacological inhibitors, natural compounds, and nanotechnology-based formulations to attenuate inflammation, preserve oral tissue integrity, and prevent alveolar bone loss by modulating inflammasome activity (Table 1).

### 4.1. Direct NLRP3 Inhibitors: MCC950 and OLT1177

Direct pharmacological inhibition of NLRP3 has demonstrated promising outcomes in preclinical studies. MCC950 (also known as CRID3) inhibits NLRP3 oligomerization, caspase-1 activation, and IL-1β maturation. It remains the most extensively characterized and effective selective NLRP3 inhibitor among current candidates [107]. In vivo experiments have shown that MCC950 significantly attenuates gingival inflammation, suppresses osteoclastogenesis, and prevents alveolar bone loss in ligature-induced periodontitis models [40]. In diabetic periodontitis models, MCC950 protects periodontal tissues by reducing mitochondrial ROS generation and inhibiting macrophage pyroptosis [108]. Genetic studies corroborate these findings: mice deficient in *NLRP3* or *caspase-1* exhibit markedly reduced alveolar and periapical bone resorption compared with WT controls [11,41]. Although the therapeutic efficacy of MCC950 has been extensively validated in preclinical OID models, its clinical translation remains limited, underscoring a critical gap between experimental success and human validation. Another direct NLRP3 inhibitor, OLT1177 (dapansutrile), currently under clinical evaluation for systemic inflammatory disorders, has shown robust NLRP3-specific inhibitory activity [94]. However, its role in oral inflammatory disease contexts remains largely unexplored. OLT1177 may hold therapeutic potential for periodontal treatment. The translational applicability of both agents could be enhanced through localized drug delivery strategies, including intrapocket gels, biodegradable scaffolds, and nanoparticle-based formulations [109].

### 4.2. Natural Compounds and Antioxidants

Several naturally derived compounds with antioxidant and anti-inflammatory properties have been identified to suppress NLRP3 activation by inhibiting NF-κB signaling, reducing mitochondrial ROS accumulation, and stabilizing lysosomal membranes. Curcumin, resveratrol, epigallocatechin-3-gallate (EGCG), and quercetin have been shown to inhibit NLRP3 inflammasome assembly, decrease IL-1β and IL-18 secretion, and attenuate inflammation, indicating their potential as adjunctive, low-toxicity therapeutic agents [95]. In a rat model of periodontitis, EGCG reduced bone loss by downregulating NLRP3 and NF-κB expression in periodontal tissues [96], whereas curcumin improved PDL integrity by upregulating the anti-inflammatory cytokine IL-10 and suppressing IL-1β and IL-6 expression [97]. A systematic review further confirmed that curcumin consistently decreases pro-inflammatory cytokines (IL-1β, IL-6, and TNF-α) and MMPs while enhancing anti-inflammatory mediators, reinforcing its translational potential as an adjunctive periodontal therapy [110]. The stability, bioavailability, and therapeutic efficacy of these phytochemicals are substantially improved through nanoparticle-based formulations, which also enhance their anticancer effects [111].

### 4.3. Cytokine Blockade: IL-1 and IL-18 Inhibitors

Targeting downstream cytokines (IL-1β and IL-18) produced via NLRP3 inflammasome activation, offers a promising therapeutic approach. Biologic agents such as anakinra (an IL-1 receptor antagonist), canakinumab and gevokizumab (anti–IL-1β monoclonal antibodies), and tadekinig-alfa (an IL-18 binding protein) have demonstrated efficacy in systemic inflammatory disorders by mitigating cytokine-mediated tissue injury [99,100,101]. Although clinical data regarding OID remain limited, preclinical studies suggest that these biologics can attenuate periodontal inflammation and tissue destruction [112,113]. Moreover, localized cytokine blockade may reduce the systemic adverse effects associated with conventional administration routes and enhance the overall safety and specificity of therapeutic interventions for OID.

### 4.4. Emerging Therapies and Nanotechnology-Based Strategies

Recent therapeutic strategies focus on modulating upstream triggers of NLRP3 activation, including mitochondrial dysfunction, ionic flux dysregulation, and excessive ROS accumulation. Rapamycin and its analogs are among the key agents that enhance autophagy-mediated clearance of inflammasome activators, decrease ROS levels, and suppress NLRP3 inflammasome signaling through the p38 mitogen-activated protein kinase and -NFκB pathways [114,115]. A major advancement involves macrophage-targeted nanotherapeutics, such as GLU@MCC, which selectively target pro-inflammatory M1 macrophages and have demonstrated enhanced efficacy in reducing alveolar bone resorption and cellular apoptosis in chronic periodontitis models [103]. Precision-based molecular approaches, including small interfering RNA (siRNA) and clustered regularly interspaced short palindromic repeats (CRISPR)–mediated gene silencing of NLRP3, provide additional avenues for the specific modulation of inflammasome activity [104]. Moreover, hydrogel- and nanocarrier-based platforms facilitate sustained local delivery of anti-inflammatory agents, offering considerable promise as next-generation therapeutic strategies within the challenging oral microenvironment [116].

## 5. Discussion

This review consolidates current evidence on the multifaceted role of the NLRP3 inflammasome in major oral inflammatory diseases and underscores its emerging potential as a therapeutic target. Consistent with prior research, the findings reaffirm that dysregulated NLRP3 activation drives IL-1β and IL-18 release, pyroptosis, and immune dysregulation, thereby promoting the initiation and progression of OID [14,31,117]. These insights collectively strengthen the hypothesis that chronic NLRP3 activation constitutes a unifying pathological mechanism underlying both soft and hard tissue destruction in the oral cavity (Table 2).

### 5.1. Interpretation Across Oral Diseases

Chronic microbial stimulation, extracellular and intracellular DAMPs, and mechanical stress exerted by orthodontic or prosthodontic appliances activate the NLRP3 inflammasome. This activation induces the secretion of pro-inflammatory cytokines IL-1β and IL-18 in gingival epithelial cells, fibroblasts, and macrophages, contributing to epithelial degradation through GSDMD–mediated pyroptosis [21,23,59,60]. These cytokines further recruit inflammatory cells, stimulate MMP activity, and enhance prostaglandin E (PGE) synthesis, which collectively contribute to periodontal tissue destruction and alveolar bone resorption [25,30]. Although the NLRP3 inflammasome is well recognized for promoting osteoclastogenesis in infection-induced periodontitis [118], recent evidence suggests a more context-dependent role. Under sterile conditions, NLRP3-driven pyroptosis can suppress osteoclast differentiation in macrophages [43], indicating that the influence of NLRP3 on bone biology may vary depending on the surrounding microenvironment. During pulpitis, the pulp chamber becomes exposed to the oral cavity following tooth fractures or carious lesions. Microbial invasion through dentinal tubules enables NLRP3 sensing within the previously sterile pulp tissue. The confined and hypoxic conditions of the pulp microenvironment amplify NLRP3 activation in odontoblasts, fibroblasts, and pulp-resident immune cells [44,48]. This process contributes to persistent inflammation and pain and, if untreated, promotes the progression of periapical lesions and alveolar bone loss [52,53]. Notably, pharmacological inhibition of NLRP3 has been shown to significantly mitigate these pathological changes in vivo [45].

Activation of the NLRP3 inflammasome during orthodontic force application plays a key role in OTM. A recent study demonstrated that OTM was significantly delayed in NLRP3^−/−^ mice compared with WT mice, emphasizing the functional significance of NLRP3 in bone remodeling and tooth displacement [63]. A controlled, transient NLRP3 response facilitates physiological bone remodeling and supports desired tooth movement [119]. However, uncontrolled or prolonged mechanical force, or the coexistence of poor oral hygiene, amplifies NLRP3 signaling and upregulates RANKL production in PDL fibroblasts and osteoblasts, accelerating pathological bone resorption [61,62]. In prosthodontic tooth replacement, titanium corrosion particles and dysbiotic biofilms surrounding dental implants induce lysosomal stress and trigger strong NLRP3 activation, contributing to peri-implantitis—a process mechanistically similar to inflammation associated with orthopedic implants [2,58,60].

In oral cancer, elevated NLRP3 and IL-1β expression frequently correlate with tumor progression, largely driven by mitochondrial dysfunction, impaired autophagy, and alterations in the tumor-associated microbiota [74]. In OSCC, persistent exposure to irritants and chronic mucosal inflammation repeatedly activate the NLRP3 inflammasome [73]. Consequently, NLRP3-induced cytokines accelerate angiogenesis, mediate immune suppression, promote EMT, and foster a tumor-permissive microenvironment that advances OSCC progression [13,74]. Although certain studies report that NLRP3 exhibits antitumor effects by inducing pyroptosis or enhancing CD8^+^ T-cell infiltration [87], most evidence indicates that NLRP3 supports OSCC cell migration, invasion, and proliferation [77]. Similarly, a strong association between the NLRP3 inflammasome and OLP has been established in several studies [71]. However, reduced NLRP3 activity has been shown to impair antifungal immunity against *Candida* infection [68]. In contrast, in Sjögren’s syndrome, NLRP3 activation within salivary gland epithelial cells promotes IL-1β–mediated acinar cell injury and contributes to xerostomia and glandular dysfunction [88,89].

Collectively, these findings demonstrate that excessive or dysregulated NLRP3 activation in response to DAMPs and PAMPs contributes to chronic inflammation and tissue destruction in major OID. Nonetheless, the NLRP3 inflammasome may exert conditional protective effects under specific circumstances, warranting further clarification through well-controlled animal model studies.

### 5.2. Therapeutic Implications

The pivotal role of the NLRP3 inflammasome in OID makes it an attractive therapeutic target. Direct inhibitors, such as MCC950 and OLT1177, suppress NLRP3 oligomerization, prevent inflammasome assembly, and attenuate inflammatory cytokine production, thereby limiting osteoclastogenesis, mucosal inflammation, and alveolar bone loss in preclinical models [94,107]. Additionally, targeting downstream cytokines IL-1β and IL-18 using agents such as IL-1ra or IL-18 binding proteins effectively reduces inflammation and tissue destruction by blocking NLRP3-driven cytokine maturation [96]. Alternatively, several natural compounds—including catechins, curcumin, EGCG, resveratrol, and quercetin—modulate NLRP3 activation through antioxidant mechanisms and inhibition of microbial virulence mediated by TLR/NF-κB signaling, providing tissue-protective effects and potential as safe adjuncts to conventional therapies [95,96,97,110].

Beyond pharmacological inhibition, advancements in precision delivery technologies, such as macrophage-targeted nanotherapeutics, biodegradable hydrogels, intrapacket gels, and nanoparticle-encapsulated formulations, enable controlled and localized drug release directly at inflammatory sites [103,113]. Although these methods minimize systemic exposure, challenges remain in overcoming oral physiological barriers such as salivary fluid turnover and biofilm penetration, which can reduce drug bioavailability [120]. Furthermore, emerging gene-silencing approaches—including siRNA and CRISPR-based technologies—offer highly specific modulation of NLRP3 activity, though they are still confined to experimental stages [104]. Notably, while microbial dysbiosis initiates disease, inflammation often persists even after bacterial reduction, suggesting that host immune dysregulation continues to drive tissue destruction [121]. Therefore, pre-clinical investigations are needed to determine whether combining standard antimicrobial or periodontal therapies with NLRP3 inhibitors or IL-1β blockers yields greater anti-inflammatory and bone-preserving efficacy than either treatment alone.

While NLRP3 inhibitors, caspase-1/IL-1 blockade, antioxidants, and phytochemicals are the focus of therapeutic approaches, recent advances suggest that preventive measures such as improved oral hygiene and oral microbiome modulation may reduce microbial-driven priming and activation signals and thereby mitigate OID [122,123]. Importantly, the oral cavity enables local delivery of anti-inflammatory agents, microbiome modulators, or NLRP3-targeted compounds via toothpaste, mouth rinses, gels, chewing sticks, or lozenges, allowing high local concentrations with reduced systemic exposure [124]. Such formulations are already being investigated for polyphenols, flavonoids, antiseptics, and probiotics, which exhibit immunomodulatory and microbiome-regulating properties. Together, these preventive and therapeutic strategies highlight the feasibility of locally modulating NLRP3 activity to manage periodontitis, peri-implantitis, and other oral inflammatory conditions.

Collectively, these findings illustrate a rapidly evolving therapeutic landscape for OID management. Although the therapeutic benefits of NLRP3 inhibition have been extensively validated in preclinical models, clinical evidence in humans remains scarce. Continued development of precision-targeted therapies is essential to achieve more personalized and durable treatment outcomes.

### 5.3. Future Perspectives and Research Priorities

Despite considerable advances in understanding the role of the NLRP3 inflammasome in OID, its clinical applicability and therapeutic translation in humans remain limited. Most existing studies have been conducted in in vitro or in animal models, underscoring the urgent need for rigorously designed human clinical trials to evaluate the safety, efficacy, and optimal dosing of NLRP3 inhibitors, IL-1β blockers, and natural anti-inflammasome agents. Longitudinal and multicenter studies are therefore crucial for establishing clinical relevance, optimizing therapeutic regimens, and advancing personalized treatment strategies. Although both experimental and clinical data strongly support the pathogenic involvement of the NLRP3 inflammasome in OID, the tissue-specific molecular mechanisms that regulate its interaction with other innate immune signaling pathways remain insufficiently characterized.

Investigating NLRP3 activity in diverse cell types—including gingival fibroblasts, dental pulp cells, oral epithelial cells, immune cells, and osteoblasts—will deepen understanding of its spatiotemporal regulation during disease onset and progression. Emerging technologies such as single-cell RNA sequencing, spatial transcriptomics, and bioengineered three-dimensional tissue models (e.g., organoids and “pulp-on-a-chip” systems) now provide powerful tools for mapping inflammasome-related cellular interactions and identifying new therapeutic targets. Integrating pharmacological inhibitors, natural modulators, cytokine-targeted agents, and nanotechnology-based delivery platforms offers a comprehensive framework for managing inflammation-driven oral diseases. Addressing these knowledge gaps is essential to fully harness the therapeutic potential of NLRP3-targeted strategies in oral and systemic health care.

## 6. Literature Search and Methodology

To identify relevant studies addressing the role of the NLRP3 inflammasome in oral and dental inflammatory diseases and its therapeutic potential, a comprehensive literature search was performed using the PubMed database (https://pubmed.ncbi.nlm.nih.gov/?otool=idkdnlblib; last accessed on 10 December 2025). To ensure the inclusion of foundational concepts, current advances, and novel therapeutic developments, the search covered publications from January 2010 to December 2025. The search strategy combined terms related to “NLRP3 inflammasome,” “oral inflammation,” “periodontitis,” “pulpitis,” “periapical lesions,” “salivary gland disorders,” “oral squamous cell carcinoma,” “orthodontic and prosthodontic complications,” and “therapeutic targeting of NLRP3.” Peer-reviewed publications—including systematic and narrative reviews as well as preclinical or translational studies—were included if they described molecular mechanisms, clinical relevance, regulatory processes, therapeutic modulation, or public health implications concerning the NLRP3 inflammasome in OID. Case reports, conference abstracts, and non-English-language articles were excluded.

## 7. Conclusions

The NLRP3 inflammasome plays a central role in the initiation and progression of major OID, including periodontitis, pulpitis, periapical lesions, orthodontic and prosthodontic treatment complications, OLP, OSCC, and SS. Dysregulated activation of NLRP3 by PAMPs and DAMPs within the oral epithelial, periodontal, and pulpal microenvironments leads to mucosal inflammation, connective tissue degradation, and alveolar bone loss. Accumulating preclinical evidence indicates that therapeutic modulation of NLRP3—through selective small-molecule inhibitors, cytokine blockade, natural anti-inflammasome compounds, and precision drug-delivery systems—can effectively suppress inflammation, preserve bone and soft tissue integrity, and slow disease progression. However, well-designed human clinical trials, biomarker-guided approaches, and a deeper understanding of the cell-specific and context-dependent regulation of NLRP3 are essential to achieve successful clinical translation. Overall, NLRP3-targeted therapeutic strategies hold substantial promise for controlling chronic oral inflammation, preserving tissue architecture, and improving long-term patient outcomes.

## Figures and Tables

**Table 1:** Summary of therapeutic strategies targeting the NLRP3 inflammasome in oral inflammatory disease. ↓: decreased.

Strategy Category	Representative Agents	Mechanism of Action	Key Benefits	Ref.
1. Direct NLRP3 Inhibitors	MCC950 (CRID3), OLT1177 (dapansutrile).	Block NLRP3 oligomerization, inhibit NLRP3 assembly formation, prevent caspase-1 activation,↓ IL-1β/IL-18/GSDMD maturation.	Block stress-induced inflammasome activation, inflammation and cell pyroptosis (gingival and pulpal), suppress osteoclastogenesis, soft tissue and peri-implantitis, and alveolar bone loss.	[40,44,45,52,94]
2. Natural Compounds and Antioxidants	Curcumin, resveratrol, EGCG, quercetin.	Suppress PAMPs-mediated TLR/NF-κB signaling; reduce ROS; inhibit NLRP3 assembly,↓ IL-1β/IL-18/GSDMD	Anti-inflammatory and antioxidant benefits, reduced tissue inflammation/ulceration, improved PDL integrity, and reduced bone loss.	[95,96,97,98]
3. Cytokine Blockade	Anakinra, canakinumab, gevokizumab, IL-18bp.	Blocks IL-1 receptor signaling and neutralizes IL-1β or IL-18.	It mitigates periodontal inflammation and suppresses mucosal damage and osteoclastogenesis.	[99,100,101,102]
4. Emerging and Nanotechnology-Based Therapies	GLU@MCC nanodrug, siRNA-NLRP3, CRISPR, rapamycin analogs.	Target M1 macrophages, Silence NLRP3 gene expression, and provide sustained local anti-inflammatory release.	↓ NLRP3–p38–NF-κB pathways, ↓ inflammatory cytokines, ↓ bone resorption and apoptosis, modulate tumor inflammation; potentially inhibit OSCC progression.	[45,103,104,105,106]

**Table 2:** Overview of NLRP3 inflammasome activation and its consequences in oral and dental diseases. ↓: decreaed; ↑: increased; →: downstream signal.

Oral/Dental Condition	Key Cellular Sources	Primary Triggers of NLRP3 Priming and Activation	Major Downstream Effects	Pathological Outcomes	Ref.
1. Periodontitis	Gingival epithelial cells, gingival fibroblasts, PDL fibroblasts, and macrophages.	Microbiome, PAMPs (LPS) → Priming. DAMPs (DC particles, ATP, ROS, mitochondrial dysfunction, K^+^ efflux, and lysosomal rupture) → Activation.	↑ IL-1β/IL-18, NET formation, M1 polarization, Th17/Treg imbalance, MMPs activation, and GSDMD-mediated pyroptosis.	Epithelial layer dysfunction, connective tissue degradation, loss of PDL attachment, PD pocket formation, and alveolar bone resorption.	[10,20,23,24,25,26,27,28,29,30,31,32,33,34,35,36,37,40,41,42,43]
2. Pulpitis and Periapical Lesions	Odontoblasts, pulp fibroblasts, endothelial cells, dendritic cells, macrophages.	Bacterial invasion → Priming. Pulp chamber (hypoxia, metabolic stress), odontoblast death–derived DAMPs (ATP, HMGB1) → Activation.	↑ IL-1β/IL-18,↑chemokine (CCL2, CXCL8), ↑MMPs, neutrophil infiltration, nociceptor activation, GSDMD-dependent pyroptosis.	Drives inflammatory response in pulp, Pulp cell necrosis, hyperalgesia, ↓ tertiary dentin. ↑ RANKL, apical periodontitis, periapical bone resorption.	[44,45,46,47,48,49,50,51,52,53,54,55,56]
3. Orthodontic/Prosthodontics appliance induced inflammation	PDL cells, osteoblasts, macrophages, and neutrophils.	PAMPS, TLR signaling → Priming. Mechanical compression, hypoxia, ATP release, ROS accumulation, titanium/metal ions, and lysosomal damage → Activation.	IL-1β/IL-18 production, ↑ RANKL, osteoclast activation, inflammasome-mediated sterile inflammation.	Orthodontic pain, external root resorption, delayed healing, periodontal inflammation. Peri-implantitis, peri-implant mucositis, denture stomatitis.	[57,58,59,60,61,62,63,64,65,66]
4. Oral Mucosal Diseases (e.g., OLP, RAS, OLK)	Oral keratinocytes, T cells, macrophages.	Microbial dysbiosis, PAMPS → Priming. *Candida*-associated DAMPs, cellular stress, galectin-3 signaling, chemical irritants → Activation.	IL-1β/IL-18 release, immune cell recruitment, epithelial damage, pyroptosis.	Mucosal ulceration, erosion, epithelial barrier dysfunction, chronic inflammation in OLP. Possible role in RAS and OLK (needs further study).	[67,68,69,70,71,72]
5. Oral Cancer (OSCC)	Keratinocytes, stromal cells, tumor-associated macrophages (TAMs).	Microbial PAMPs → Priming. Chemical carcinogens (tobacco, alcohol), DAMPs from necrotic tumor cells, ROS, mtDNA, metabolic stress → Activation.	IL-1β/IL-18-driven angiogenesis, EMT activation, TAMs recruitment, Tumor progression, GSDMD-mediated pyroptosis (context dependent).	Tumor growth, invasion, metastasis, poor prognosis. Some antitumor effects via pyroptosis or CD8+ T-cell activation.	[73,74,75,76,77,78,79,80,81,82,83,84,85,86,87]
6. Salivary Gland Disorders (Sjögren’s Syndrome)	Acinar cells, gland/ductal epithelial cells, macrophages.	Autoantigen–immune complexes, mitochondrial dysfunction, ROS accumulation, P2X7R activation, DNA damage-associated DAMPs.	IL-1β-driven acinar apoptosis, chronic lymphocytic infiltration, fibrosis.	Xerostomia, glandular atrophy, chronic sialadenitis.	[88,89,90,91,92,93]

## Data Availability

No new data were created or analyzed in this study. Data sharing is not applicable to this article.

## Abbreviations

The following abbreviations are used in this manuscript:

OID	Oral inflammatory diseases
LRR	Leucine-rich repeat
NLRP3	Nucleotide-binding oligomerization domain, leucine-rich repeat, and pyrin domain-containing 3
PAMPs	Pathogen associated molecular patterns
DAMPs	Danger associated molecular patterns
IL	Interleukin
GSDMD	Gasdermin D
OSCC	Oral squamous cell carcinoma
ASC	Apoptosis-associated speck-like protein containing a caspase-1 recruitment domain
NACHT	Nucleotide-binding and oligomerization domain
PYD	Pyrin domain
CARD	Caspase activation and recruitment domain
LPS	Lipopolysaccharide
TNF-α	Tumor Necrosis Factor alpha
TLRs	Toll like receptors
NODs	Nucleotide-binding and Oligomerization Domains
NF-κB	Nuclear factor kappa-light-chain-enhancer of activated B
ATP	Adenosine tri phosphate
ROS	Reactive oxygen species
DC	Dental calculus
NET	Neutrophil extracellular traps
MMPs	Matrix metalloproteinases
PDL	Periodontal ligament
RANKL	Receptor activator of nuclear factor kappa-B Ligand
NFATc1	Nuclear factor of activated T-cells, cytoplasmic 1
CtpK	Cathepsin K
CCL2	C-C motif chemokine ligand
CXCL	C-X-C motif chemokine ligand
PGE2	Prostaglandin E2
OTM	Orthodontic tooth movement
OLP	Oral lichen planus
RAS	Recurrent aphthous stomatitis
OLK	Oral leukoplakia
TAMs	Tumor-associated macrophages
EMT	Epithelial–mesenchymal transition
SS	Sjögrens syndrome
EGCG	Epigallocatechin-3-gallate
IL-1ra	IL-1 receptor antagonist
IL-18bp	IL-18 binding protein
